# Psychoemotional Features in Irritable Bowel Syndrome

**Published:** 2012-12-25

**Authors:** D Dragoş, O Ionescu, DG Ojog, MD Tănăsescu

**Affiliations:** * "Carol Davila" University of Medicine Bucharest, 1st Internal Medicine Department, University Emergency Hospital Bucharest, Romania; ** "Carol Davila" University of Medicine Bucharest

**Keywords:** colonic functional disorders, personality inventory, psychological profile, psychological predisposition to disease

## Abstract

**Objective.** To delineate the psychological profile of individuals with irritable bowel syndrome (IBS).

**Method. **A triple questionnaire of 614 items (including psychological and medical ones) was given to 10192 respondents and the results were analyzed by means of Cronbach alpha and Chi square test, together with an ad-hoc designed method that implied ranking and outliers detecting.

**Results and conclusions.** Anxiety and depression are general psychological tendencies unspecifcally linked with IBS. Among the features with a relatively more specific correlation with IBS, tension has the strongest association, followed by the inclination to endure unacceptable situations, preoccupation for health, and susceptibility, and then by fear of failure and sense of demanding profession.

IBS individuals readily accept a subordinate position, which may be connected to their history of tyrannical parents, and also to their preoccupation for authority factors. The sense of being treated unfairly by the authority persons during the school years nuances this last feature. Some features that bring some nuances to this psychological portrait are: contemplative nature and analyzing tendency, preoccupation with health issues, a reserved, unsociable, and precautious nature, clinging to known circumstances.

**Abbreviations: **ChiSq = chi-square; OdRa = odds ratio; OdRaCL = OdRa confidence limits; ErrProb = probability of error; SS = statistically significant; CrA = Cronbach alpha; a / m = the calculations were done by taking into account the average/ maximal score; P / M = psychological / medical category; PaMm / PmMa / PmMm / PaMa = the calculations were done by taking into account the average score for the PsyCt and the maximal score for the MedCt / the maximal score for PsyCt and the average score for the MedCt / and the maximal score for both / and the average score for both; FD = functional dyspepsia; IBS = irritable bowel syndrome; IBSCt = IBS category; MedCt = medical category; PsyCt / PsyIt = psychological category / item.

## Introduction

The reasons we have embarked in the enterprise of looking for the psychoemotional associations of the various internal disorders are described in previous articles [**1,2**]. It is by means of a cross-sectional study that we are striving to reach our goal, based on a triple questionnaire of our own design (the manner we have arrived to the items of this questionnaire is presented in the first of the two above-mentioned papers).

 We also explore the possibility that the various colonic symptoms might have different psychological connotations.

 There is no need of studies exploring the psychological connotations of IBS [**3-15**] – they are reviewed in another paper of this issue [”A Review of the Psychoemotional Factors in Irritable Bowel Syndrome”]. Why starting a new one? Because the psychological disorders whose links with IBS have already been explored are too general, too broad, the result being the lack of specificity. More about this in our previous article [**1**].

## Materials and methods

The materials and methods are the same as those described in the paper about functional dyspepsia (FD) published in this issue [“Psychoemotional Features of a Doubtful Disorder: Functional Dyspepsia”]: the data analysis has gone through the same stages, the same have been the Cronbach alpha (CrA) based criteria used to establish the medical category (MedCt) we are dealing with in this paper, we have applied the same statistical exigencies, we have used the same sample.

## Results

We have used the same terms used to designate the psychological categories as in the above-mentioned paper on FD. 

**Stage 1 – the right assignment of the medical items**

 On the basis of medical significance and of item-to-item associations (quantified by CrA) (too numerous to be presented here), we divided the colonic symptoms into diarrhea-type, constipation-type, and IBS-type, and we were left with two lonely items, one asking about mucus in stools, the other about meteorism. In this paper, we present the results concerning typical IBS. Those pertaining to disorders of the intestinal transit (diarrhea and constipation) and to the two remaining colonic symptoms (mucus in stools and meteorism) are the object of two other papers in the same issue [“Psychoemotional Features Associated with Disorders of the Intestinal Transit” and “Psychoemotional Correlations of two Orphane Colonic Symptoms: Mucus in Stools and Meteorism” respectively].

**IBS items**

 The items ”I had abdominal discomfort/ pain associated with a change in stool frequency for…” and ”I have felt abdominal discomfort/ pain related to changes in stool consistency (i.e. softening or hardening of stools) for” are very strongly correlated, and both of them have reasonably good correlations with ”I have had abdominal discomfort/ pain relieved by stool for” (**Table 1**). 

**Table 1:** The best correlations of the IBS items, evaluated by means of Cronbach alpha (CrA).

	The correlated items:	CrA
”I had abdominal discomfort/ pain associated with a change in stool frequency for…”	”I have felt abdominal discomfort/ pain related to changes in stool consistency (i.e. softening or hardening of stools) for”	0.85
”I have had abdominal discomfort/ pain relieved by stool for”	”I had abdominal discomfort/ pain associated with a change in stool frequency for…”	0.67
”I have had abdominal discomfort/ pain relieved by stool for”	”I have felt abdominal discomfort/ pain related to changes in stool consistency (i.e. softening or hardening of stools) for”	0.69

Besides the above associations, these three items have no other significant correlations: the CrA for the association with any other item (including any other item referring to colonic symptoms, such as meteorism, mucus in stools, diarrhea- or constipation-type symptoms) is <0.25. Therefore, it is justified to join these three items in a single category – we have named it the “IBS category” (IBSCt). It seems to be consistent enough, as evaluated by CrA (**Table 2**). 

**Table 2:** Evaluating the internal consistency of IBSCt by means of Cronbach alpha (CrA) (“§” marks the item that lowers the group CrA).

The missing item	CrA
None	0.81
”I have felt abdominal discomfort/ pain related to changes in stool consistency (i.e. softening or hardening of stools) for”	0.67
”I had abdominal discomfort/ pain associated with a change in stool frequency for…”	0.69
§ ”I have had abdominal discomfort/ pain relieved by stool for”	0.85

Although of acceptable strength, the correlation of the third item with the first two is far from the strength of their reciprocal correlation. Unavoidably, this third item lowers the group CrA (the 2nd criterion is met only in its weak variant). Nevertheless, as the other three criteria are definitely fulfilled, we declare as valid this first category of colonic symptoms (i.e. the three items are referring to the same pathological condition).

**Stage 2 – establishing the correlation between psychological and the medical items/categories**

There are thousands of statistically significant (SS) (namely with an ErrProb <10E-07) correlations among the item-to-item ones, precluding their exhaustive presentation.

The next table (**Table 3**) displays the correlations of the IBSCt with the various PsyCts, the calculations being made by taking into account the average score for the IBSCt and the maximal score for the PsyCts.

**Table 3:**
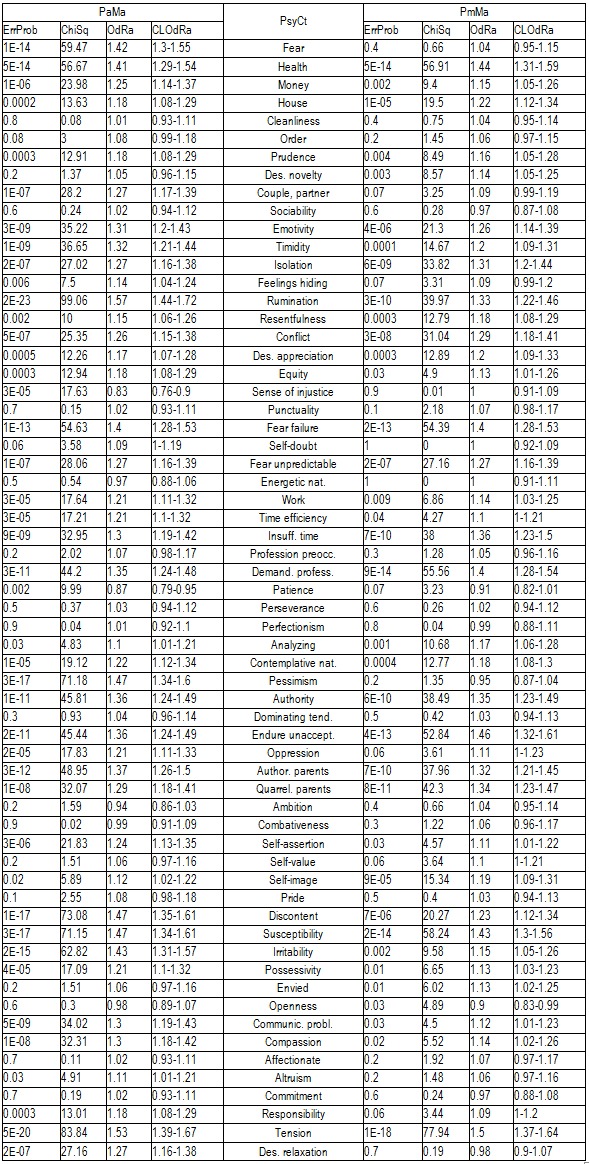
Correlation of IBSCt with the various PsyCts, taking into account the average score for IBSCt (Ma) and either the average (Pa) or the maximal (Pm) score for the PsyCts.

The following table (**Table 4**) comparatively presents the psychological correlations of IBSCt category generated by means of the other two methods of calculating: by taking into account the maximal score for IBSCt (Mm) and either the average (Pa) or the maximal (Pm) score for the PsyCts.

**Table 4:**
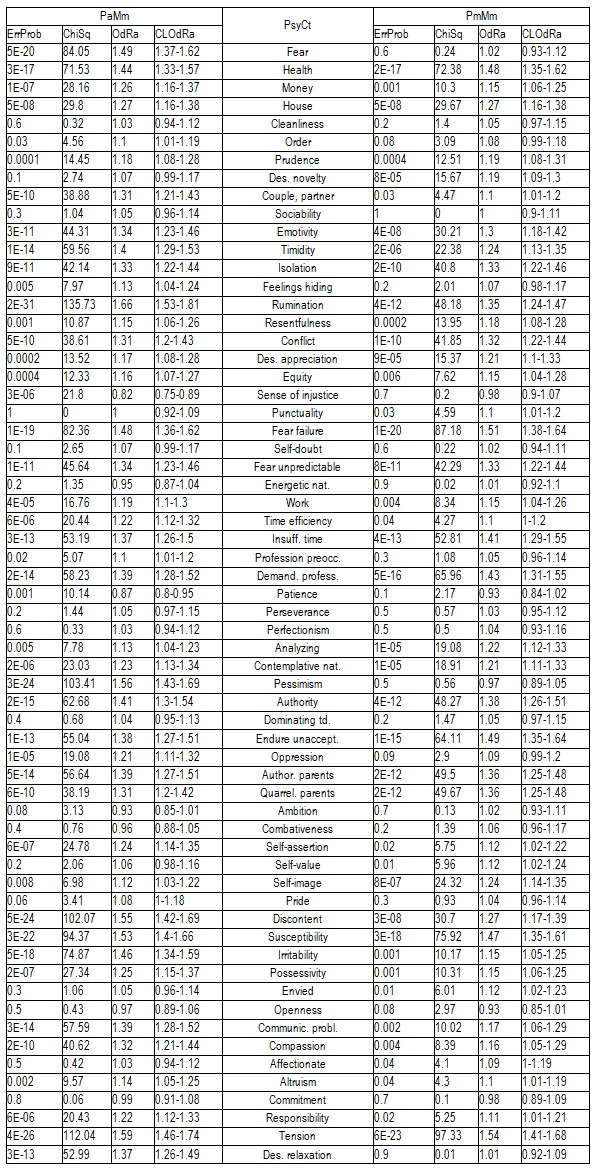
Correlation of IBSCt with the various PsyCts, taking into account the maximal score for IBSCt (Mm) and either the average (Pa) or the maximal (Pm) score for the PsyCts.

**Fig. 1:**
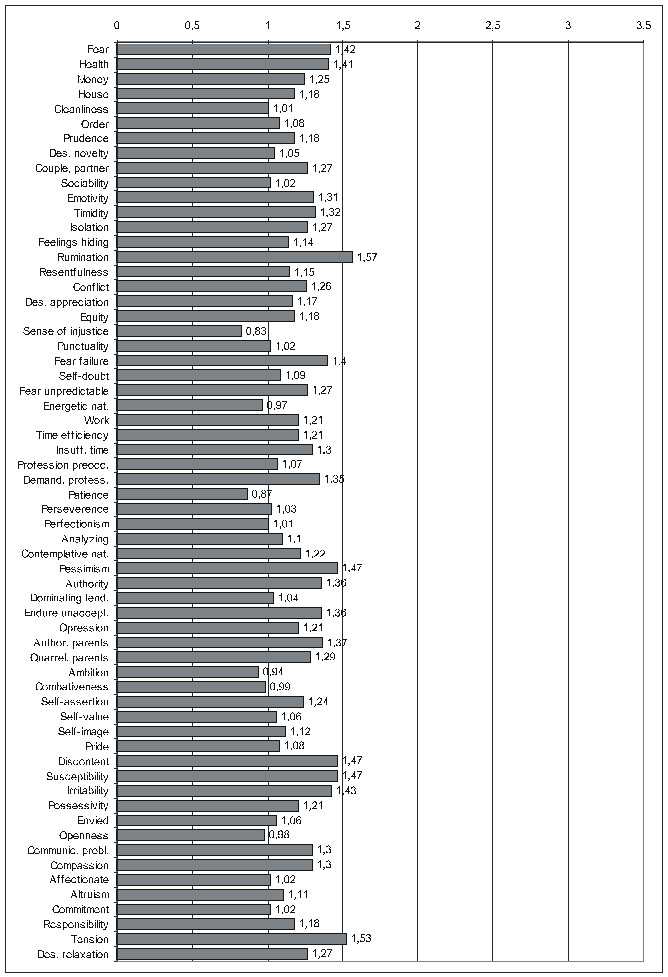
Correlation of IBSCt with the various PsyCts, taking into account the average score for both (PaMa) (the numbers labeling each bar are the odds ratio values).

**Fig. 2:**
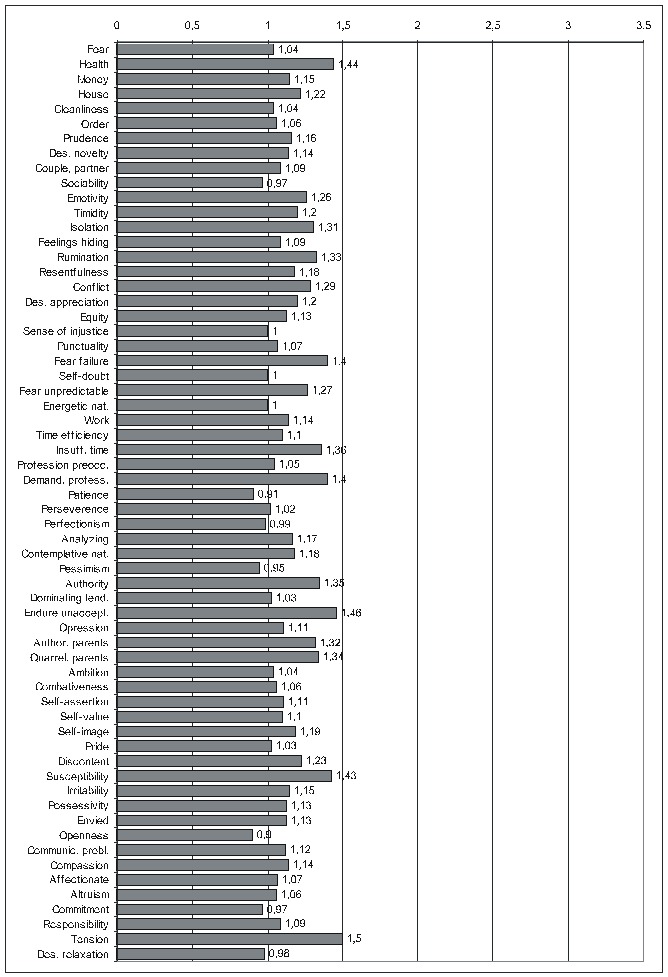
Correlation of IBSCt with the various PsyCts, taking into account the maximal score for the PsyCt and the average score for the IBSCt (PmMa) (the numbers labeling each bar are the odds ratio values).

There is not enough room to display all the diagrams corresponding to the results in the previous two tables, therefore, we shall limit ourselves to those derived from the calculations done by taking into account the average score for IBSCt and either the average (**Fig. 1**) or the maximal (**Fig. 2**)) score for the PsyCts (PaMa and PmMa respectively). The third diagram (**Fig. 3**) illustrates a revealing particularity: for several PsyCts, we obtained conspicuously higher OdRa values by using the average (“Pa”) versus the maximal (“Pm”) scores for the PsyCts, visually translated in strikingly longer top two bars than the bottom two bars in each group of four (i.e. for each PsyCt).

**Figure:**
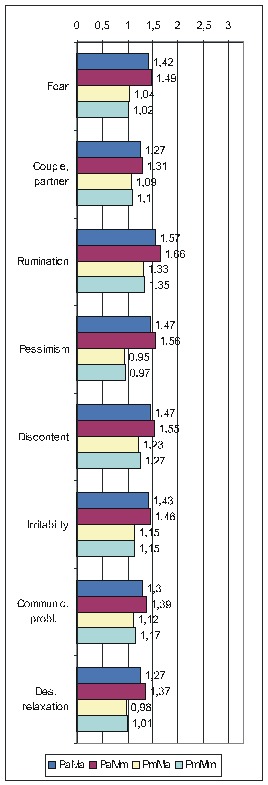
Comparative OdRa demonstrating the higher values obtained by averaging the scores for the PsyCts (Pa) (top two bars in each group of four) as compared to the those derived when the maximal scores for the PsyCts (Pm) were used in the computations (bottom two bars in each group of four) [**1**].

**Stage 3 – searching for ranking outsiders**

We shall present only the SS results. The ensuing three tables are presenting those engendered by ranking the correlations of PsyCts to the IBSCt (**Table 5**), of the PsyIts to the IBSCt (**Table 6**), and of the PsyIts to the IBS items (**Table 7**).

**Table 5:** Searching for ChiSq- and OdRa-ranking outsiders among the PsyCt-to-IBSCt correlations

Calculations method	Rank	ErrProb	ChiSq	OdRa	OdRaCL	PsyCt
PaMa, ranking by ChiSq	11	1E-05	19.12	1.22	1.12-1.34	Contemplative nat.
PaMm, PaMa, ranking by OdRa	7	0.002	9.99	0.87	0.79-0.95	Patience

**Table 6:**
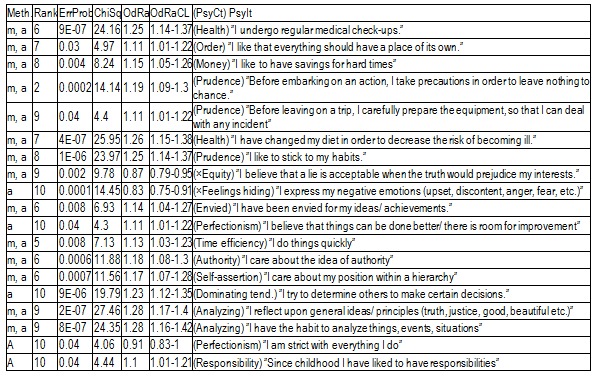
Searching for ranking outsiders among the PsyIts-to-IBSCt correlations (Meth. = method of doing the calculations, “m” / “a” = the maximal / average scores for the IBSCt were used in the calculations).

**Table 7:**
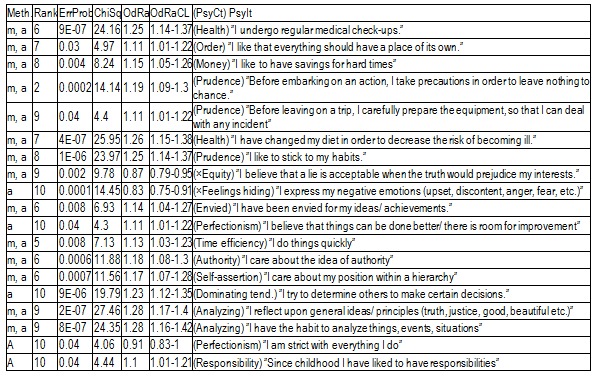
Searching for ChiSq- and OdRa-ranking outsiders among the correlations of PsyIts with IBS items (RankCrit = ranking criterion, “×” means that the opposite of that item was taken into account).

## Discussions

The psychological profile of the IBS individual (**Fig. 1**) only vaguely reminds of that associated with the other colonic disorders / symptoms [see the articles “Psychoemotional Features Associated with Disorders of the Intestinal Transit” and “Psychoemotional Correlations of two Orphane Colonic Symptoms: Mucus in Stools and Meteorism” in the same issue] – everything is flattened. 

We notice the tendency of some of the most prominent correlations to lose in magnitude when going from the results produced by averaging the scores to those obtained by using the maximal scores for the PsyCts (**Fig. 2,Fig. 3**), especially fear and pessimism. Interestingly, these are the psychological variables (better known as anxiety and depression) whose correlation with IBS is most studied. We remind our interpretation [see the paper “Psychoemotional Features of a Doubtful Disorder: Functional Dyspepsia”] that, although prominent in IBS patients, these two psychological features are not specific. The intensities of the correlations of irritability and of several other PsyCts have, at their turn, shrunk. Somehow, more specific are probably the PsyCts whose correlations with IBSCt were not altered by using the maximal scores in the calculations. Among these, tension seems to be the most prominent (while the inclination to relaxation looses any degree of correlation), followed by the tendency to endure unacceptable situations, preoccupation for health, and susceptibility. Almost equally strong are the associations with fear of failure and sense of demanding profession. The correlation with tyrannical and quarrelling parents may be linked with the association with the preoccupation for authority factors.

 The information provided by ranking the correlations is probably more specific (we have argued this in the above-mentioned paper on FD). 

 Ranking the category-to-category correlations (**Table 5**) seems to suggest a contemplative nature. There may also be a lack of patience – but the correlation is of marginal significance.

 Ranking the correlations of the IBS items to IBSCt produces only several relatively SS results, suggesting preoccupation for health (we already knew it) and analyzing tendency (which is coherent with the contemplative nature). Several items seem to indicate an inclination to be prudent (money saving, carefully preparing for potential incidents) and to adhere to familiar circumstances (objects should be left at their established places; the individual would rather follow his habits).

 The negative association with equity reminds of a characteristic of diarrhea patients [see the paper “Psychoemotional Features Associated with Disorders of the Intestinal Transit”].

 Besides these correlations, there are still others, but only marginally SS. For the moment, it is not worth speculating about them.

 Among the many item-to-item correlations (**Table 7**), there are only three absolutely SS ones. One of them (with the PsyIt ”I have the habit to analyze things, events, situations”) corroborates the analyzing and contemplative tendency, and quite a few others seem to point in the same direction. We should therefore admit this as psychological characteristic of IBS patients. Among the relatively SS correlations, we have found arguments for the preoccupation for health and lack of sociability. 

 The other absolutely SS correlation pertains to the rumination PsyCt, which has a non-negligible association with IBS, but we should probably be more specific: his/ her blunders (which he/ she probably regard as failures) are the most difficult to forget for the IBS individual.

 Finally, the third absolutely SS correlation is with an item belonging to the sense of injustice category. As this category, taken as a group, does not correlate with IBS, we should probably be quite narrow in our conclusions: IBS individuals do not have a sense of universal injustice, but are likely to consider that, in school, they were unfairly treated by the authority persons – interesting revelation, as it concurs with idea of domineering parents and with the preoccupation with authority factors. There are some weak associations that seem to point to the same idea, suggesting a propensity to accept a subordinate position and to shun competition, yet there are others that seem to contradict it (”I try to determine others to make certain decisions”).

 There are also several weak negative correlations with items of the dominating tendency category (”In a group, I’m the one who calls the tune”, ”At meetings/ parties, I make the introductions”, ”In a group I am the one who animates/ brightens the atmosphere”) (and we are inclined to believe that IBS individuals do not have a dominating nature), but they may also be interpreted as manifestations of a shy, reserved nature. The more so as there are several negative correlations with sociability items (”I have friends…”, ”I like being with my group of friends”).

## Conclusions

The associations of the traditionally recognized psychological factors (anxiety, depression) with IBS are rather unspecific. Relatively more specific is the association of IBS primarily with tension, than with the inclination to endure unacceptable situations, preoccupation for health, and susceptibility, and with fear of failure, sense of demanding profession. 

 The submissive nature is suggested by the association with PsyCts such as tyrannical parents, preoccupation for authority factors, and the sense of being treated unfairly by the authority persons during the school years.

 Other psychological particularities of the IBS patients are the contemplative nature and analyzing tendency, hypchondriacal tendency, lack of sociability, and inclination to take precautionary measures while abiding to known circumstances.
